# NAC1 attenuates BCL6 negative autoregulation and functions as a BCL6 coactivator of FOXQ1 transcription in cancer cells

**DOI:** 10.18632/aging.103203

**Published:** 2020-05-14

**Authors:** Min Gao, Alice Laschuk Herlinger, Renchin Wu, Tian-Li Wang, Ie-Ming Shih, Beihua Kong, Leticia Batista Azevedo Rangel, Jin-Ming Yang

**Affiliations:** 1 Department of Obstetrics and Gynecology, Qilu Hospital of Shandong University, Jinan, PR China; 2 Departments of Gynecology and Obstetrics, Oncology and Pathology, Johns Hopkins Medical Institutions, Baltimore, MD 21287, USA; 3 Biotechnology Program/Renorbio, Health Science Center, Federal University of Espírito Santo, Vitória, Brazil; 4 Biochemistry and Pharmacology Program, Health Science Center, Federal University of Espírito Santo, Vitória, Brazil; 5 Department of Pharmaceutical Sciences, Federal University of Espírito Santo, Vitória, Brazil; 6 Department of Toxicology and Cancer Biology, College of Medicine, Markey Cancer Center, University of Kentucky, Lexington, KY 40536, USA; 7 Department of Genetics, Federal University of Rio de Janeiro, Rio de Janeiro, RJ, Brazil

**Keywords:** NAC1, FOXQ1, BCL6, transcription, cancer

## Abstract

Background: Nucleus accumbens-associated protein 1 (NAC1) has multifaceted roles in cancer pathogenesis and progression, including the development of drug resistance, promotion of cytokinesis, and maintenance of “stem cell-like” phenotypes. NAC1 is a transcriptional co-regulator belonging to the bric-a-brac tramtrack broad (BTB) family of proteins, although it lacks the characteristic DNA binding motif of the BTB family. The formation of higher-order transcription complexes likely depends on its interaction with other DNA-binding co-factors.

Results: NAC1 interacts with BCL6 via its C-terminal BEN domain and forms a complex that binds the promoter region and activates transcription of the NAC1 target gene, FOXQ1. NAC1 and BCL6 were coordinately upregulated. Our analysis also identified a novel function of NAC1 in attenuating BCL6 auto-downregulation in ovarian cancer. Lastly, we found a significant overlap among NAC1- and BCL6-regulated genes in tumor cells, suggesting that NAC1 and BCL6 coordinately control transcription in cancer.

Conclusions: The results of this study provide a novel mechanistic insight into the oncogenic roles of NAC1 and underline the importance of developing the NAC1/BCL6-targeted cancer therapy.

Methods: Using the Cistrome database and Chromatin Immunoprecipitation (ChIP) analyses, we identified BCL6 as a potential NAC1- interacting molecule. Co-immunoprecipitation (Co-IP), luciferase reporter assay, immunohistochemistry and microarray analysis were performed to analyze the interaction between NAC1 and BCL6 and the mechanisms by which they regulate the downstream genes including FOXQ1.

## INTRODUCTION

Nucleus accumbens-associated protein 1 (NAC1) is a transcriptional co-regulator that belongs to the bric-a-brac tramtrack broad (BTB) family of proteins. Formation of higher-order transcription complexes likely depends on its interaction with other DNA-binding co-factors [1]. Like other members of the BTB family, on the other hand, homodimeric or heterodimeric interactions mediated by the BTB domain are crucial for functionality [2]. NAC1 mediates a plethora of biological functions [3–6] and has multifaceted roles in ovarian cancer pathogenesis and progression, including promotion of cell survival, proliferation and motility [2, 7–9], prevention of cell senescence [10], activation of autophagy [11], and acquisition of a chemoresistant phenotype [2, 8, 11–13].

NAC1 is overexpressed in several cancer types including cervical, endometrial, and ovarian cancers [2, 14, 15] and has been described as one of the most important driver genes in ovarian carcinogenesis [16]. Upregulation of NAC1 is associated with shortened progression-free survival and acquisition of the paclitaxel- and cisplatin-chemoresistance [2, 8, 11, 12, 17], and inhibiting the homodimerization of NAC1 protein can sensitize cancer cells to anticancer agents [18]. Moreover, NAC1 regulates the expression of over 700 genes in ovarian cancer, including FOXQ1, which is associated with increased mobility of tumor cells [19]. Nevertheless, the precise role of NAC1 in FOXQ1 transcription remains to be elucidated.

We hypothesize that NAC1 regulates gene expression through formation of a transcription complex with BCL6, another BTB family member [20]. BCL6 protein represses its own transcription in a corepressor-dependent manner by binding to sites within exon 1 of the *BCL6* gene [21]. In diffuse B-cell lymphoma, a translocation within the 5’ regulatory region of *BCL6* has been linked to disruption of its negative autoregulation, leading to its anomalous upregulation and possible involvement in pathogenesis [22, 23]. BCL6 is also overexpressed in breast and colorectal cancers [24–26]. Moreover, in ovarian cancer, BCL6 expression is closely correlated with FIGO staging, lymph node metastasis and recurrence and has been recently described as a negative prognostic factor. BCL6 induces cell proliferation, migration and invasion when overexpressed in ovarian cancer cell lines [27].

We have found that BCL6 is a co-factor in NAC1-containing transcription complexes. NAC1 interacts with BCL6 via its C-terminal BEN domain and forms a transcription complex that binds to the promoter region of the NAC1 target gene, FOXQ1. Moreover, we showed that NAC1 and BCL6 are coordinately upregulated in ovarian cancer, and NAC1 plays an important role in attenuating BCL6 autoregulation. Lastly, we observed a significant overlap among NAC1- and BCL6-regulated genes in tumor cells. The results of this study provide a novel mechanistic insight into the oncogenic roles of NAC1, and underlines the importance of developing NAC1/BCL6-targeted cancer therapy.

## RESULTS

### NAC1/BCL6 transcription complex modulates the expression of FOXQ1 and other NAC1 target genes

**Figure 1:**
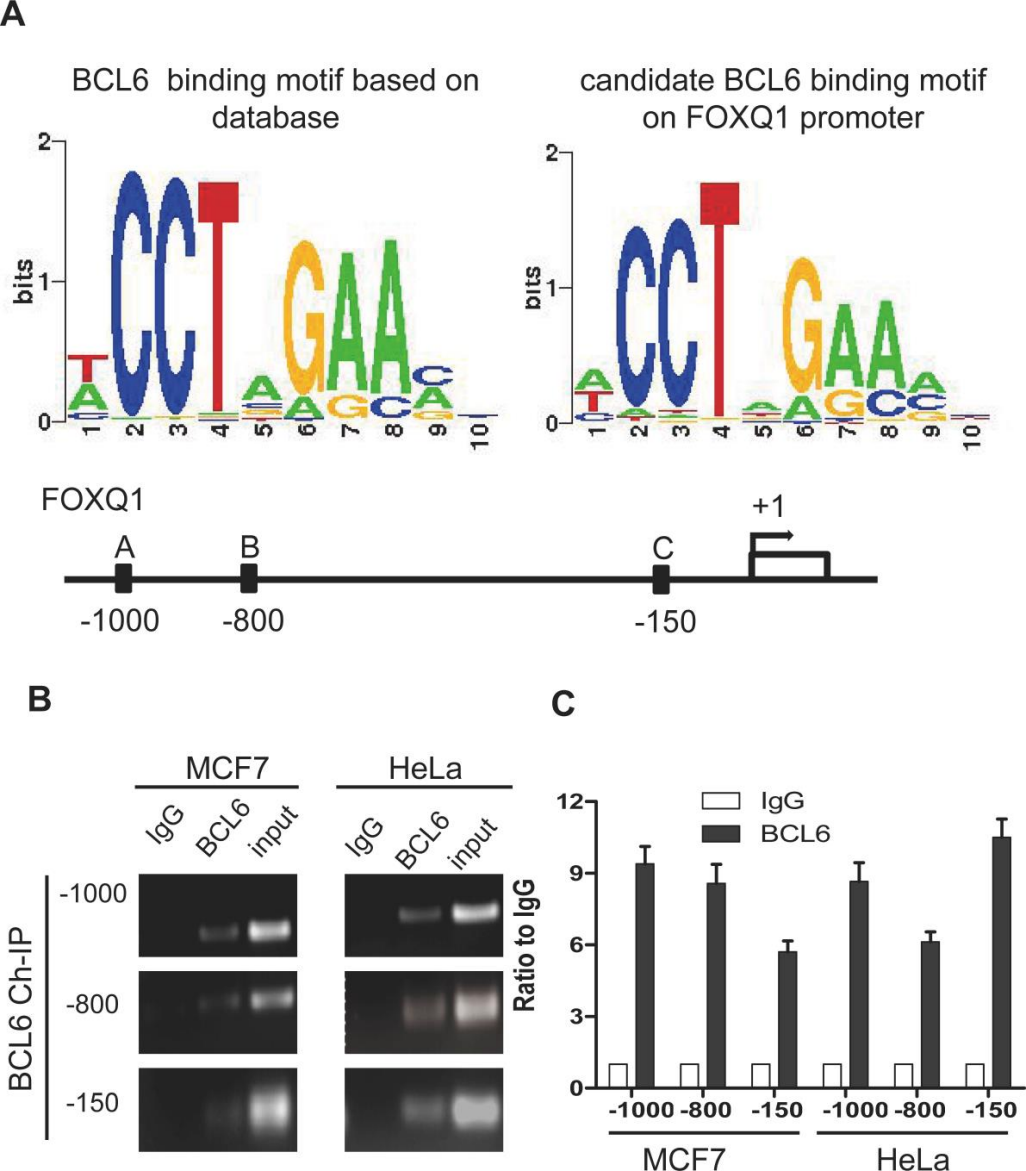
**FOXQ1 promoter has BCL6 binding motifs.** (**A**) The BCL6 consensus motif was retrieved with Cistrome analysis and the position frequency matrix of BCL6 motif is presented in the left panel. On the right panel the consensus sequence of BCL6 binding motifs on FOXQ1 promoter resulting from the Cistrome analysis is represented. Below, a schematic map was provided to show Positions A/B/C of FOXQ1 promoter loci. (**B**) Chromatin immunoprecipitation with anti-BCL6 and IgG antibodies in MCF7 and HeLa cells. (**C**) Semi-quantitative PCR was used to evaluate the enrichment of the three different putative BCL6 binding motifs of the *FOXQ1* promoter.

Because NAC1 lacks the hallmark DNA binding motif of the BTB protein family, its capacity to modulate the expression of NAC1-target genes depends on interactions with other DNA-binding co-factors to form higher-order transcription complexes [1]. To identify potential NAC1- interacting molecules in the transcription complexes, we analyzed the transcription factor binding sites within promoter regions of the NAC1-regulated genes previously reported by our group [19]. We found that the BCL6 consensus binding motif mapped to the promoters of several of these genes including *FOXQ1*, a gene directly regulated by NAC1. Previously, we demonstrated that NAC1 is essential and sufficient for activation of FOXQ1 transcription and that the role of NAC1 in cell motility is mediated, at least in part, by FOXQ1. NAC1 knockdown decreased FOXQ1 expression and promoter activity. In silico analysis revealed a significant co-up-regulation of NAC1 and FOXQ1 in ovarian carcinoma tissues. We identified three putative BCL6 binding motifs within the *FOXQ1* promoter region at positions A (-1000), B (-800), and C (-150) upstream of the transcription start site (Figure 1A). To determine whether BCL6 binds to these three identified consensus sites within the *FOXQ1* promoter region, we conducted ChIP experiments using the cell lines, HeLa and MCF7, known to express abundant NAC1 [4, 10, 19]. BCL6-associated chromatin fragments could be specifically immunoprecipitated with anti-BCL6 rabbit polyclonal antibody but not with the rabbit IgG isotype control (Figure 1B). To confirm that the DNA fragment immunoprecipitated by the anti-BCL6 antibody contains the putative BCL6 binding motifs located in the *FOXQ1* promoter region, we performed qPCR with three different primer sets flanking the candidate motifs at positions A (-1000), B (-800), and C (-150) (see Table 1). Each primer set amplified the expected motif selectively from the BCL6 ChIP but not from the control ChIP with P< 0.01 for all three motifs (Figure 1C), suggesting that *FOXQ1* expression is directly modulated by BCL6. Thus, BCL6 is a candidate co-factor in NAC1-containing transcription complexes.

**Table 1:** – Oligonucleotide primers sequences.

**Primer**	**Application**	**Sequence**
NAC1 F	qPCR	5’-AAGCTGAGGATCTGCTGGAA-3’
NAC1 R	qPCR	5’-CCAGACACTGCAGATGGAGA-3’
FOXQ1 F	qPCR	5’-CTCAACGACTGCTTCGTCAA-3’
FOXQ1 R	qPCR	5’-GTGTACTCGCTGTTGGGGTT-3’
BCL6 F	qPCR	5’-TGGGGTTCTTAGAAGTGGTGA-3’
BCL6 R	qPCR	5’-CAATGCCTTGCTTCACAGTC-3’
APP F	qPCR	5’-AAAGAACTTGTAGGTTGGATTTTCG-3’
APP R	qPCR	5’- GTGAAGATGGATGCAGAATTCCG-3’
GAPDH F	qPCR	5’-TTGGTATCGTGGAAGGACTC-3’
GAPDH R	qPCR	5’-ACAGTCTTCTGGGTGGCAGT-3’
FOXQ1(-1000) F	ChIP	5’-ATCCAAAAAGGCGCATACAA-3’
FOXQ1(-1000) R	ChIP	5’-AGAAATTGCGCAGGACACTT-3’
FOXQ1(-800) F	ChIP	5’-CGTAAGCAGCATCGTTTTCA-3’
FOXQ1(-800) R	ChIP	5’-CCTAGGGGGACACCTGAAG-3’
FOXQ1(-150) F	ChIP	5’-ACATCATCCGGCACCATT-3’
FOXQ1(-150) R	ChIP	5’-CTCGTTAAAGAGCCCAGGAG-3’
BCL6(227-421) F	ChIP	5’-GGGTTCTTAGAAGTGGTG-3’
BCL6(227-421) R	ChIP	5’-CAAAGCATTTGGCAAGAG-3’
MouseBCL6 F	qPCR	5’-AAAGGCCGGACACCAGTTTT-3’
mouseBCL6 R	qPCR	5’-AACGTCCGTCAAGATGTCCC-3’
mouseGAPDH F	qPCR	5’-AGGTCGGTGTGAACGGATTTG-3
mouseGAPDH R	qPCR	5’- GGGGTCGTTGATGGCAACA-3

**Figure 2:**
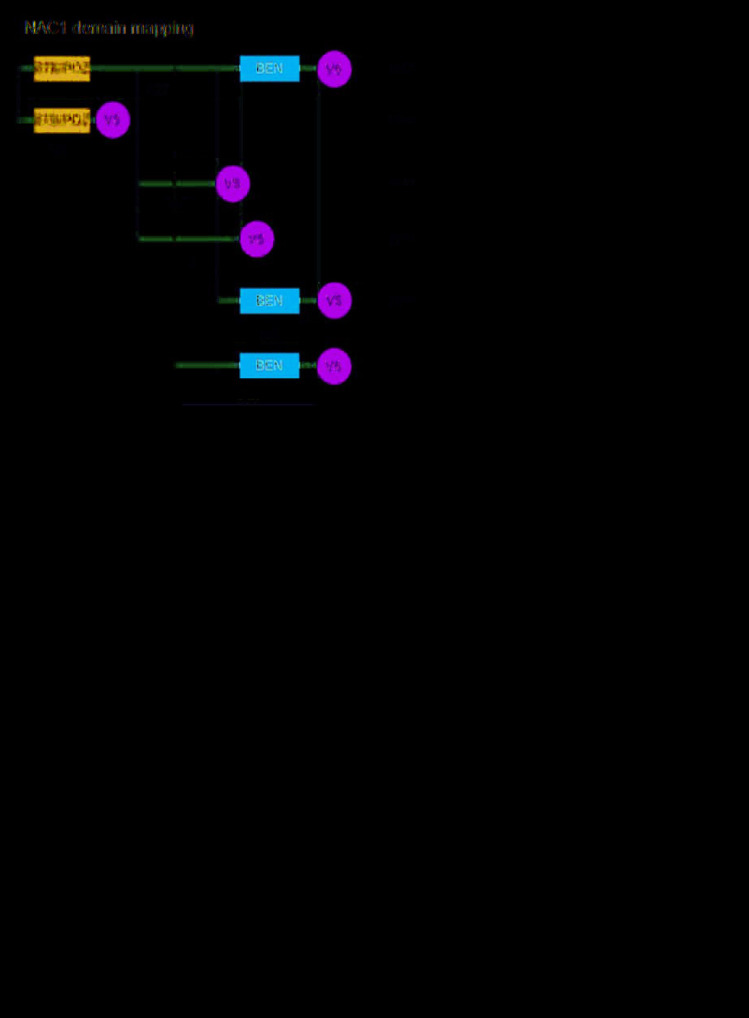
**Co-immunoprecipitation (co-IP) analysis of interactions of NAC1 with BCL6.** (**A**) Representation of the NAC1 domain mapping corresponding to the different NAC1 partial constructs used. (**B**) HEK293T cells were transfected with NAC1-V5 and BCL6-Flag constructs, and the lysates were collected for reciprocal protein co-IP experiments. (**C**) Reciprocal co-IP of full-length NAC1 and NAC1- deletion mutants with BCL6.

We then asked whether NAC1 binds BCL6 to form a transcription complex that regulates the expression of NAC1 target genes such as *FOXQ1*. We transfected HEK293T cells with constructs containing the coding sequences of BCL6-Flag and with either full-length NAC1-V5 or each of the five NAC1 deletion mutants (Figure 2A). Results from reciprocal co-immunoprecipitation experiments conducted in HEK293T cells co-transfected with a BCL6-Flag plasmid and a full-length NAC1-V5 plasmid showed that NAC1 directly interacted with BCL6 (Figure 2B). We then sought to map the NAC1 domain necessary for the interaction with BCL6. We co-transfected HEK293T cells with a BCL6-Flag plasmid and each of the NAC1 fragments N129, M147, M171, C186, and C250. Whereas the first three NAC1 fragments failed to immunoprecipitate with BCL6-Flag, C186 and C250, which encompass the 186 and 250 C-terminal amino acids of NAC1, were immunoprecipitated with BCL6-Flag, suggesting that the C-terminal region is sufficient for the interaction between NAC1 and BCL6 (Figure 2C). Both of the fragments contain the BEN domain, an alpha-helical module that mediates protein-DNA and protein-protein interactions during chromatin organization and transcription [28]. These data indicate that the NAC1 C-terminal BEN domain is essential for the interaction of NAC1 with BCL6 to form a transcription complex, which likely binds to specific promoter regions of NAC1 target genes.

### Transcriptional activity of FOXQ1 promoter is triggered by a NAC1-dependent BCL6-binding mechanism

**Figure 3:**
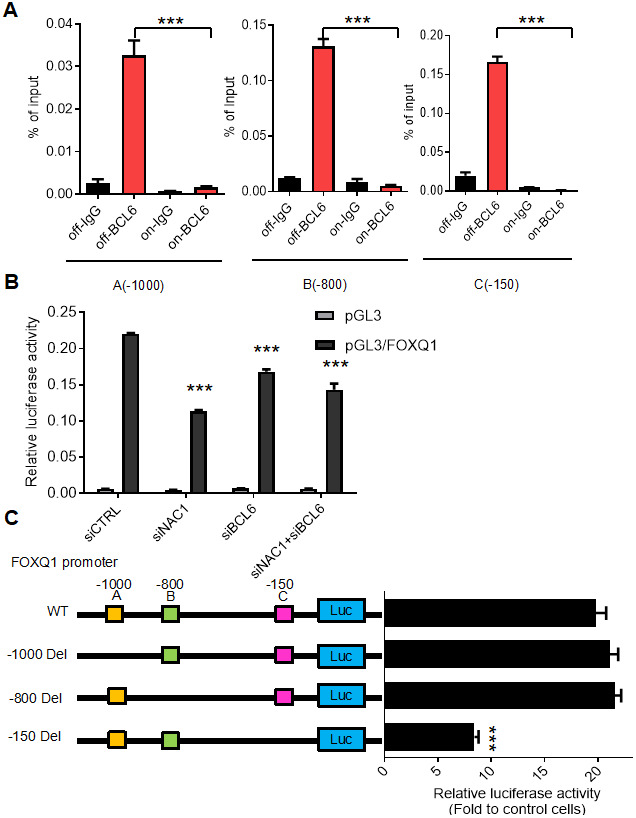
**BCL6 activates *FOXQ1* promoter activity in a NAC1-dependent fashion.** (**A**) Chromatin immunoprecipitation of BCL6 in HeLa tTa cells expressing active NAC1 (off) and after NAC1 inactivation by N130 truncated NAC1 expression induction (on). qPCR with three different primer sets flanking three different regions, which contain the putative BCL6 binding motifs of *FOXQ1* promoter: A (-1000), B (-800), and C (-150). BCL6 binding to each region is represented as percentage of the input. (**B**) Luciferase reporter assay of *FOXQ1* promoter activity in HeLa cells transfected with NAC1 and BCL6 specific siRNA. Data show relative luciferase activity normalized to Renilla luciferase activity from each experimental condition; (**C**) HeLa cells were transfected with BCL6-Flag or control plasmid. The relative luciferase activities of wide type FOXQ1 promoter and three deletion mutant promoters (FOXQ1-del A -1000, del B -800, and del C -150) were determined. ***P<0.001.

To determine the requirement for a functional NAC1 in the putative NAC1/BCL6 complex, we assessed the transcription of *FOXQ1* in HeLa N130 tTA cells. HeLa N130 tTA cells express a normal NAC1 when grown in the presence of doxycycline (system off), but express a truncated, dysfunctional NAC1 (N130) that combines with and inactivates endogenous NAC1 when cultured in the absence of doxycycline (system on) [2]. We found that, when the system was off (presence of dox), there was significant co-immunoprecipitation of BCL6; in contrast, when the system was on (absence of dox), the induction of N130 (leading to loss of functional NAC1) resulted in a significant decrease (P< 0.001) in the abundance of BCL6 co-immunoprecipitated with the three BCL6 binding motifs within the *FOXQ1* promoter (Figure 3A). To further evaluate the critical contribution of NAC1 and BCL6 in the expression of *FOXQ1*, we conducted a luciferase reporter assay of *FOXQ1* promoter activity in HeLa cells whose expression of NAC1 or/and BCL6 were silenced by specific siRNAs. The results showed that the activity of *FOXQ1* promoter was significantly reduced (P< 0.01) when the expression of NAC1, BCL6, or both genes was suppressed (Figure 3B) (for knock-down efficiencies, see Supplementary Figure 1). We next assessed the contribution of the three putative BCL6 binding motifs within the FOXQ1 promoter region at positions A (-1000), B (-800), and C (-150). We constructed three mutant reporter plasmids with deletion of each position and examined luciferase activity in BCL6-overexpression cells. Deletion of the upstream of FOXQ1 transcription start site 150 nucleotides (position C) resulted in decreased luciferase activity as compared with the original sequence in BCL6-knockdown cells. Other mutants with deletions in two other sites (position A and B) did not show reduced luciferase activity (Figure 3C). These results indicate that binding sites in the -150 region upstream of the transcription start site is responsible for transcriptional regulation of FOXQ1 by BCL6. Thus, we propose a mechanism by which NAC1 and BCL6 interact and collaborate to regulate *FOXQ1* transcriptional activity.

### NAC1 and BCL6 are co-upregulated in ovarian cancer

**Figure 4:**
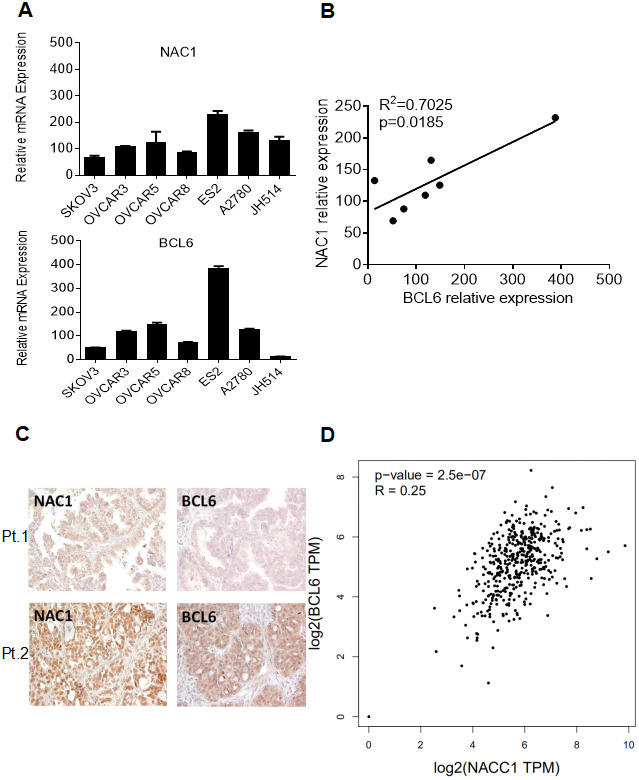
**Co-expression of NAC1 and BCL6.** (**A**) qPCR was performed on a panel of cell lines to detect the levels of NAC1 and BCL6 mRNA. (**B**) Correlation analysis between NAC1 and BCL6. (**C**) Immunohistochemistry analysis was performed to detect the protein expression levels of BCL6 and NAC1 in serous high-grade ovarian carcinoma tissues. Two patients results were shown as examples. (**D**) The co-expression of NAC1 and BCL6 was verified using the TCGA datasets.

NAC1 has been appreciated to play an important role in ovarian carcinogenesis and cancer progression [2, 8, 11, 19]. Our observation that NAC1 and BCL6 activate *FOXQ1* promoter activity via a positive, cooperative mechanism suggest that these molecules may be coordinately regulated in ovarian cancer. To test this hypothesis, we examined the expression of NAC1 and BCL6 transcripts in a panel of 7 ovarian cancer cell lines and assessed NAC1 and BCL6 protein expression in 51 high-grade serous ovarian adenocarcinoma samples (Figure 4A and 4C). Importantly, we observed a strong correlation between the expression of *NACC1* and *BCL6* transcripts in the ovarian cancer cell lines tested (R^2^=0.7025; P=0.0185) (Figure 4B). Moreover, NAC1 and BCL6 protein expression in ovarian cancer samples showed a similar pattern (R^2^=0.31, P=0.027; graph not shown). Furthermore, the correlation between NAC1 and BCL6 expression was verified using the GEPIA (retrieval date, August 22, 2019), which is based on a large sample cohort from TCGA databases (R^2^=0.25; P=2.5e-07) (Figure 4D). All of these observations support a coordinate up-regulation of NAC1 and BCL6, and underscore the importance of the NAC1/BCL6 complex in ovarian cancer.

### NAC1 interacts with BCL6 and prevents its auto-downregulation

The negative auto-regulation of *BCL6* transcription relies on its interaction with other BTB/POZ corepressors [21]. In view of our finding that NAC1 and BCL6 are co-upregulated in ovarian cancer specimens and cell lines, we wanted to test the possibility that NAC1 modulates the expression of BCL6. Therefore, using both overexpression and knockdown approaches we investigated the role of NAC1 in the BCL6-mediated auto-downregulation.

**Figure 5:**
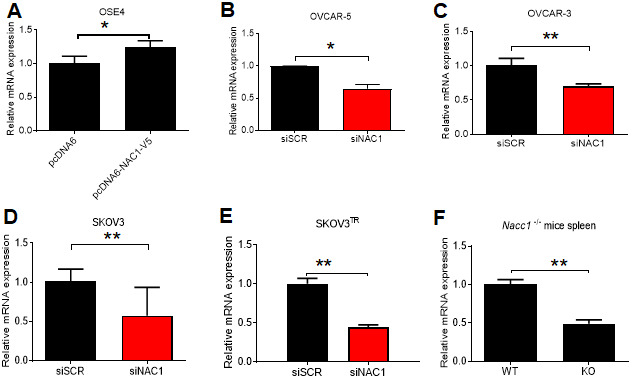
**NAC1 expression modulates BCL6 transcription.** (**A**) OSE4 cells were transfected with NAC1-V5 constructs. BCL6 expression was assessed by qPCR 48 h post-transfection. NAC1-targeting siRNA was transfected into OVCAR-5 (**B**), OVCAR-3 (**C**), SKOV3 (**D**), and SKOV3^TR^ (**E**) cells 48h prior to analysis of BCL6 expression by qPCR. (**F**) BCL6 mRNA expression was assessed in Nacc1-/- mouse spleen tissue. *P<0.05, **P<0.01.

We induced ectopic overexpression of NAC1 by transfecting OSE4 cells with V5-tagged NAC1. Overexpression of NAC1 resulted in a significant increase in the transcription of *BCL6* after 48 h (P=0.041) (Figure 5A). In OVCAR-5 cells with silencing of NAC1 expression, we observed a significantly reduced *BCL6* transcription (P=0.016) (Figure 5B). Similar reduction of *BCL6* transcription was observed in OVCAR-3 (P=0.0089), SKOV3 (P=0.0216) cells, and in the taxane-resistant derivate line SKOV3^TR^ (P=0.0002), as compared to the cells transfected with a control siRNA (Figure 5C–5E). Of relevance, the expression of *BCL6* transcript was reduced by 50% in the spleen of *Nacc1*^-/-^ mice (P=0.0068) (Figure 5F). NAC1 expression levels and efficiencies of siRNA knockdown are presented in Supplementary Figure 2.

**Figure 6:**
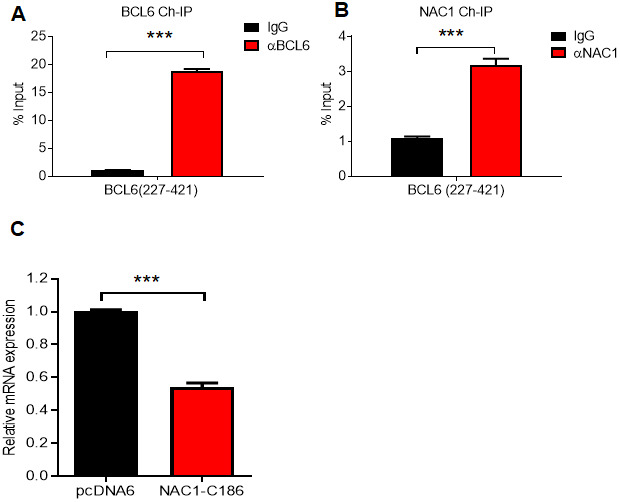
**NAC1 binding to BCL6 is necessary to prevent BCL6 auto-downregulation.** Chromatin immunoprecipitation of BCL6 (**A**) and NAC1 (**B**) in OVCAR-3 cells. qPCR was performed with a primer set targeting the BCL6 autoregulation binding-site, which is located in BCL6 exon 1 (BCL6 227-421). (**C**) Competition assay analysis of BCL6 downregulation in OVCAR-3 cells 48 h after ectopic expression of NAC1-C186; ***P<0.001.

We next inquired whether the modulation of BCL6 expression by NAC1 is dependent on the interaction between these two molecules. To answer this question, we first performed an endogenous NAC1 and BCL6 ChIP on the consensus BCL6 binding sequence within the *BCL6* promoter in ovarian cancer cells. The results showed that BCL6 bound to its own promoter, and more interestingly, NAC1 was enriched in the chromatin immunoprecipitate (Fig. 6A and 6B). Then, we used a competition assay in which the expression of BCL6 was assessed in OVCAR-3 cells ectopically expressing the NAC1 C-terminal 186 amino acids (NAC1-C186), which comprise the minimum NAC1 domain capable of binding BCL6. We found that *BCL6* expression decreased by approximately 50% when cells were transformed with NAC1-C186-V5, in comparison with the cells transfected with the control construct pcDAN6 (P<0.0001) (Figure 6C). These data imply that a direct interaction between NAC1 and BCL6 plays a key role in attenuating the BCL6 auto-downregulation by NAC1.

### NAC1 and BCL6 coordinately regulate gene expression in OVCAR-5 cells

**Figure 7:**
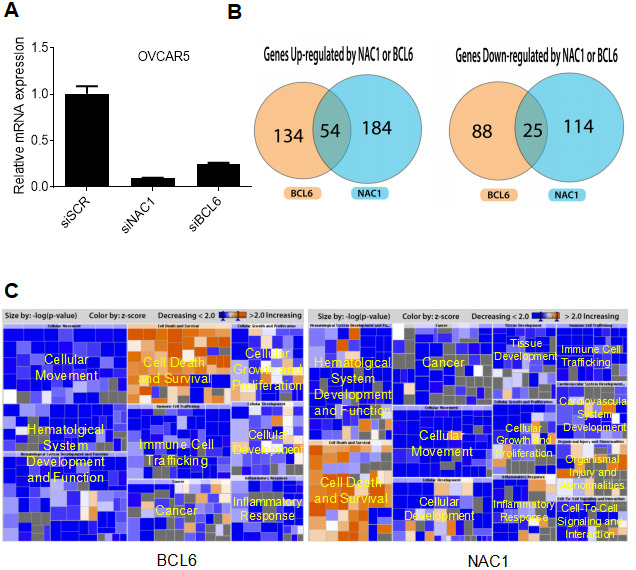
**NAC1 and BCL6 regulated genes.** (**A**) qPCR was performed to determine the robustness of siRNA-mediated NAC1 and BCL6 knockdown. (**B**) Venn diagrams showing genes up- or down-regulated by NAC1 and BCL6. (**C**) Pathway analysis of genes affected by knockdown of NAC1 (right panel) or of BCL6 (left panel).

To identify the NAC1/BCL6 transcription-complex target genes, we performed transcriptome analysis in OVCAR-5 cells that constitutively overexpress NAC1 and BCL6. We compared transcriptional profiles of cells transfected with a NAC1 siRNA, BCL6 siRNA, or control siRNA. The efficiency of knockdown was tested by qPCR 72 h after transfection. There was a significant down-regulation of NAC1 and BCL6 in the cells transfected with their respective targeting siRNAs in comparison with the control siRNA (P<0.0001) (Figure 7A). Following NAC1 or BCL6 knockdown, 238 and 188 genes were down-regulated respectively, indicating that the expression of these genes is positively regulated by NAC1 and BCL6. Up-regulation of 139 and 113 genes were observed in the NAC1- and BCL6-knockdown cells, indicating that these genes were negatively regulated by NAC1 and BCL6. Among these, a group of 54 genes were down-regulated and a group of 25 genes were up-regulated in both NAC1- and BCL6-knockdown cells (Figure 7B). Pathway analysis of genes modulated by NAC1 (Figure 7C, right panel) and BCL6 (Figure 7C, left panel) in OVCAR-5 cells indicates that the genes involved in cell death and survival are the most intensively up-regulated, and the genes that participate in cell proliferation and movement are largely down-regulated by NAC1 and BCL6 (Figure 7C, Supplementary Table 1). In addition, we found a significant overlap of NAC1- and BCL6-regulated genes in OVCAR-5 cells, suggesting a transcription collaboration of NAC1 and BCL6 in ovarian cancer cells.

## DISCUSSION

Although the functions of NAC1 are mainly mediated through its transcriptional regulatory activity, the precise mechanisms remain incompletely understood [19], partly because NAC1 lacks a characteristic DNA binding motif of the BTB protein family [1]. Here, we demonstrate that NAC1 and BCL6 directly interact to form a transcription complex, and that NAC1 plays a key role in modulating BCL6 transcriptional activity.

**Figure 8:**
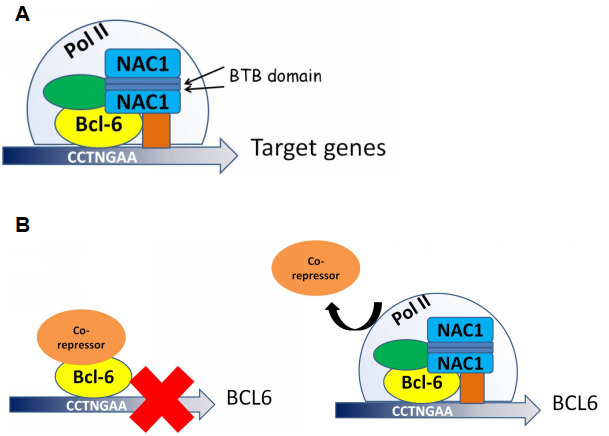
**Proposed models for NAC1 and BCL6 activation of transcription and NAC1 attenuation of BCL6 negative autoregulation**. (**A**) NAC1 forms a homodimer by interaction of its BTB domains. NAC1 homodimer interacts with BCL6 protein through the 186 amino acids from its C-terminus. NAC1-BCL6 interaction forms a higher magnitude complex that regulates transcription of target genes. (**B**) When NAC1 is absent, BCL6 protein is complexed with its co-repressors, which inhibits BCL6 transcription. When upregulated, NAC1 displaces BCL6 corepressors, and forms a regulatory complex with BCL6, which allows BCL6 expression.

Though the interaction between NAC1 and BCL6 and their roles in gene expression modulation have been reported [20], we demonstrate here that these molecules directly interact via the BEN domain located at the 186 amino acid C-terminal portion of NAC1. We also demonstrate that NAC1 and BCL6 collaborate in regulating the transcriptional activity of *FOXQ1*. Indeed, BCL6 binds to all three putative BCL6 binding motifs within the *FOXQ1* promoter. Binding of BCL6 to these motifs depends on NAC1, as the expression of the truncated NAC1 clone N130 (leading to loss of functional NAC1) prevents BCL6 from binding to *FOXQ1* promoter, and downregulations of both NAC1 and BCL6 result in reduced *FOXQ1* promoter activity. Chromatin immunoprecipitation sequencing (ChIP-seq) might help define a more detailed mechanism underlying the coordinated transcriptional control of all downstream target genes by NAC1 and BCL6. Based on the results of this study, we propose a mechanism in which NAC1 and BCL6 form a regulatory complex and collaborate to regulate transcription factors including FOXQ1 (Figure 8A).

NAC1 overexpression has been reported in various malignancies including ovarian cancer [9]. It was shown that BCL6 was overexpressed in ovarian cancer, promoting proliferation, migration and invasion of tumor cells [27]. Prompted by our finding that NAC1 and BCL6 are co-transcriptional regulators, we investigated their expression patterns in a panel of ovarian cancer cell lines and HGSC specimens, using qPCR and immunohistochemistry respectively. We found that these two molecules were coordinately up-regulated in ovarian cancer both *in vitro* and *in vivo*, suggesting an important role of these proteins in disease development and/or progression. The similar observations were made in the cells expressing either NAC1 or BCL6 supporting that the NAC1/BCL6 transcription regulation complex may be responsible for promoting development of ovarian cancer. Overexpression of NAC1 or BCL6 has been reported to correlate with shorter disease free survival [9, 27]. Moreover, the genome-wide transcription analysis performed in this study revealed 79 genes that are regulated by both NAC1 and BCL6. Noteworthily, many of these are associated with carcinogenesis and cancer progress and participate in cell cycle progression, cell survival, and angiogenesis. Even broader roles for NAC1 and BCL6 might exist in ovarian cancer, as ingenuity pathway analysis has revealed the upregulation of IL-17, IL-8, and IL-6 signaling pathways, which were reported to be correlated with novel functions in driving tumor growth and stemness [29–31].

Auto-regulation of BCL6 expression depends upon its interaction with BTB/POZ family members. This study identified a new mechanism by which NAC1 binds to BCL6 and attenuates BCL6 auto-downregulation, thus upregulating the expression levels of BCL6 in ovarian cancer cells (Figure 8B). We propose that the co-upregulation of NAC1 and BCL6 works in concert as a transcription complex to modulate an array of their downstream genes that play critical roles in the malignant phenotype of ovarian cancer. As BCL6 has been well characterized as an oncogene [22, 32], our results may have important implications in cancer treatment or prevention.

## CONCLUSIONS

Taken together, this study establishes a new mechanism of how NAC1 acts as a driver gene in ovarian cancer. The demonstration that NAC1 promotes tumor progression at least in part by forming molecular partnership with BCL6 should further our understanding on the intricate transcription network operated in ovarian cancer and provide a molecular foundation for designing new therapeutic strategy through targeting NAC1/BCL6 transcription complex.

## MATERIALS AND METHODS

### Cell lines and culture conditions

Cell lines used in this study, HeLa, SKOV3, NIH/OVCAR-3, NIH/OVCAR-5, MCF7 and HEK293T were purchased from ATCC (Manassas, VA, USA), and were maintained in RPMI-1640 media (Invitrogen, Waltham, MA, USA) supplemented with 10% (v/v) fetal bovine serum (Cellgro, Manassas, VA, USA) and penicillin/streptomycin (Invitrogen). SKOV3^TR^ is a paclitaxel -resistant cell line grown in the presence of 0.25 μM paclitaxel [8]. HeLa N130 tTA cell line comprises a dysfunctional NAC1 system generated by the expression of the BTB domain (N130) of NAC1 induced by the removal of doxycycline from the culture media [2].

### Nacc1 knock-out mice

Mice were housed and handled according to approved protocol (MO12M405) and guidelines of the IACUC of Johns Hopkins University. *Nacc1*^-/-^ mice were obtained as described by our group [4]. Mice were euthanized, and spleens were collected for analysis.

### Promoter analysis

Promoter sequences were retrieved with the use of Biostrings and BSgenome packages of Bioconductor 2.12 [33]. The position frequency matrix of BCL6 motif (Transfac ID: M01171) was obtained from Cistrome (http://cistrome.org/~jian/motif_collection/databases/Cistrome/pwm/M01171.pwm), and was then converted to a position weight matrix with background base frequencies calculated according to different promoter sequences [34]. Occurrences of BCL6 motifs on the promoter sequences of genes potentially modulated by BCL6 were identified by the use of the function matchPWM () in the Biostrings package with the parameter minimum score = 70%. The log-odds scores of each occurrence of a BCL6 motif were calculated by the function PWMscoreStartingAt () in the Biostrings package.

### Chromatin immunoprecipitation (ChIP)

ChIP extract preparation followed a previously described method with minor changes [35]. Briefly, target cells were seeded on 150 mm dishes and cultured until 90% confluence. Cells were fixed with 1% (w/v) paraformaldehyde prior to cross-linking with DTBP. Rabbit polyclonal antibodies raised against BCL6 (SC-858; Santa Cruz Biotechnology, Dallas, TX, USA) and NAC1 (SC-98638; Santa Cruz Biotechnology), or a rabbit IgG isotype (Abcam, Cambridge, MA, USA) were used for immunoprecipitations. The precipitated DNA was used for quantitative or semi-quantitative PCR. The primers used are summarized in Table 1.

### Quantitative real-time RT-PCR (qRT-PCR)

RNA was extracted using RNAeasy kit (Qiagen, Germantown, MD, USA). First strand cDNA was synthesized using the iScript cDNA synthesis kit (Bio-Rad, Hercules, CA, USA). Relative transcript expression levels were measured using the CFX96 Real-Time PCR Detection System (Bio-Rad), and quantified by fluorescence intensity of SYBR Green I (Invitrogen). The primers used in this study are summarized in Table 1. Averages of the threshold cycle number (Ct) of triplicate measurements were obtained. The relative gene expression level was calculated by differences in Ct between the gene of interest and control genes, amyloid precursor protein (APP) or glyceraldehyde 3-phosphate dehydrogenase (GAPDH), which were used as internal references for data normalization.

### Co-immunoprecipitation (Co-IP)

HEK293T cells were used for Co-IP as previously described by our group [2], but using 1% (v/v) NP-40 lysis buffer. For V5-tagged proteins, anti-V5 agarose beads (Sigma, St Louis, MO, USA) were used. For FLAG tagged proteins, cell lysates were precipitated with anti-FLAG M2 affinity gel (Sigma). Laemmli buffer (Bio-Rad) supplemented with beta-mercaptoethanol was used to elute proteins. Proteins were analyzed by Western blot.

### Luciferase reporter assay

HeLa cells were first transfected with NAC1-targeting and/or BCL6-targeting siRNA or control siRNA. After 24 h, cells were transfected with control pGL3 plasmid or pGL3-FOXQ1 promoter firefly luciferase constructs, and the pRL-Renilla reporter plasmid (Promega, Madison, WI, USA) using Lipofectamine LTX and Plus Reagent (Invitrogen). The firefly and renilla luciferase activities were measured 24 h after transfection using a luminometer (PerkinElmer, Waltham, MA) and the Dual-Glo luciferase reagent (Promega). Firefly luciferase activity was normalized to the renilla luciferase activity.

### Immunohistochemistry

A total of 51 formalin-fixed and paraffin-embedded ovarian high-grade serous carcinoma samples were obtained from the Department of Pathology at the Johns Hopkins Hospital, Baltimore, Maryland. Paraffin tissues were arranged in tissue microarrays (TMA) to ensure that tissues were stained under the same conditions. An in-house mouse monoclonal anti-NAC1 antibody, the specificity of which has been previously confirmed by Western blotting [2], was used. A mouse anti-BCL6 monoclonal antibody was purchased from Santa Cruz (SC-858). Citrate-based Target Retrieval Solution (DAKO, Carpentaria, CA, USA) was used for antigen retrieval (95-100°C, 20 min). TMA slides were incubated overnight with the primary antibody at 4°C, and for 30 min with the secondary antibody at room temperature. Colorimetric development was detected using the EnVision+System (DAKO). The slides were counterstained with hematoxylin, and the immunoreactivity was scored independently by two investigators based on the intensity of staining, using a scale of 0 to 3+.

### Validation of the co-expression genes

To further confirm the co-expression of NAC1 and BCL6 gene, we performed an analysis using GEPIA (http://gepia2.cancer-pku.cn/; retrieval date, August 22, 2019), an online software based on the TCGA databases [36].

### Microarray analysis of gene expression

OVCAR-5 cells were transfected with either NAC1 siRNA, BCL6 siRNA, or control siRNA, and were harvested at 72 h. Total RNA was isolated using the RNeasy Plus Mini Kit (Qiagen), and RNA samples were hybridized onto the Human HT-12 v4 Expression BeadChip (Illumina, San Diego, CA, USA). By comparing global gene expression profiles among siNAC1-treated, siBCL6-treated and control cells, we prioritized a set of genes for analysis with a false discovery rate <0.002 at either time point. Genes that were upregulated or downregulated were defined using the regulation status of the time point with the lowest FDR. Genes with a >1.5-fold change compared with the control group were further analyzed by the Ingenuity Pathways Analysis System (https://www.ingenuity.com) for downstream effects analysis. This analysis is based on expected causal effects between input genes and biological functions. The expected causal effects are derived from the literature compiled in the Ingenuity Knowledge Base, and allow a prediction for each function based on the pattern of gene expression modulation.

### Statistical analyses

Two-tailed t-test was used to determine the significance of differences between groups. Data are presented as means ± SD. P<0.05 was considered statistically significant.

### Ethics approval

Mice were housed and handled according to approved protocol (MO12M405) and guidelines of the IACUC of Johns Hopkins University. This work involving human samples has been approved by The Ethics Committee of the Johns Hopkins University. Written informed consent was obtained from all the human participants involved in the study.

## Supplementary Material

Supplementary Figures

Supplementary Table 1
