# Supplementary material for: NAC1 attenuates BCL6 negative autoregulation and functions as a BCL6 coactivator of FOXQ1 transcription in cancer cells
Source: Aging (Albany NY). 2020 May 14;12(10):9275–91. doi: 10.18632/aging.103203 (PMC7288929; doi:10.18632/aging.103203)
Supplement: Supplementary Figures [file aging-12-103203-s001..pdf]

SUPPLEMENTARY FIGURES

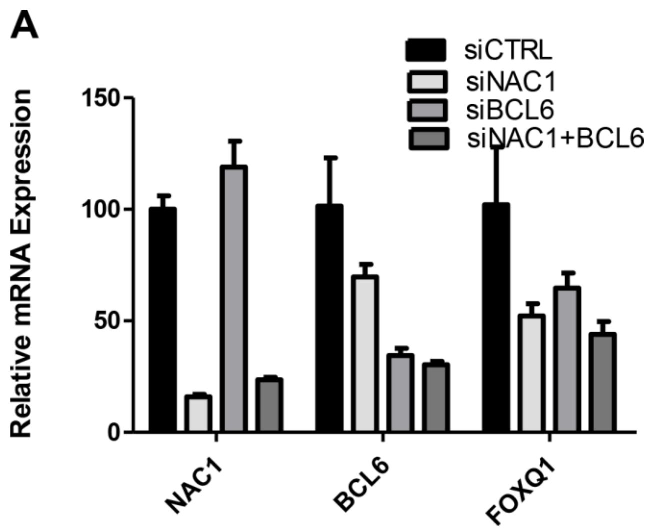

Supplementary Figure 1. Knock-down efficiencies of NAC1, BCL6, and FOXQ1 in HeLa cells transfected with NAC1 and BCL6 specific siRNA.

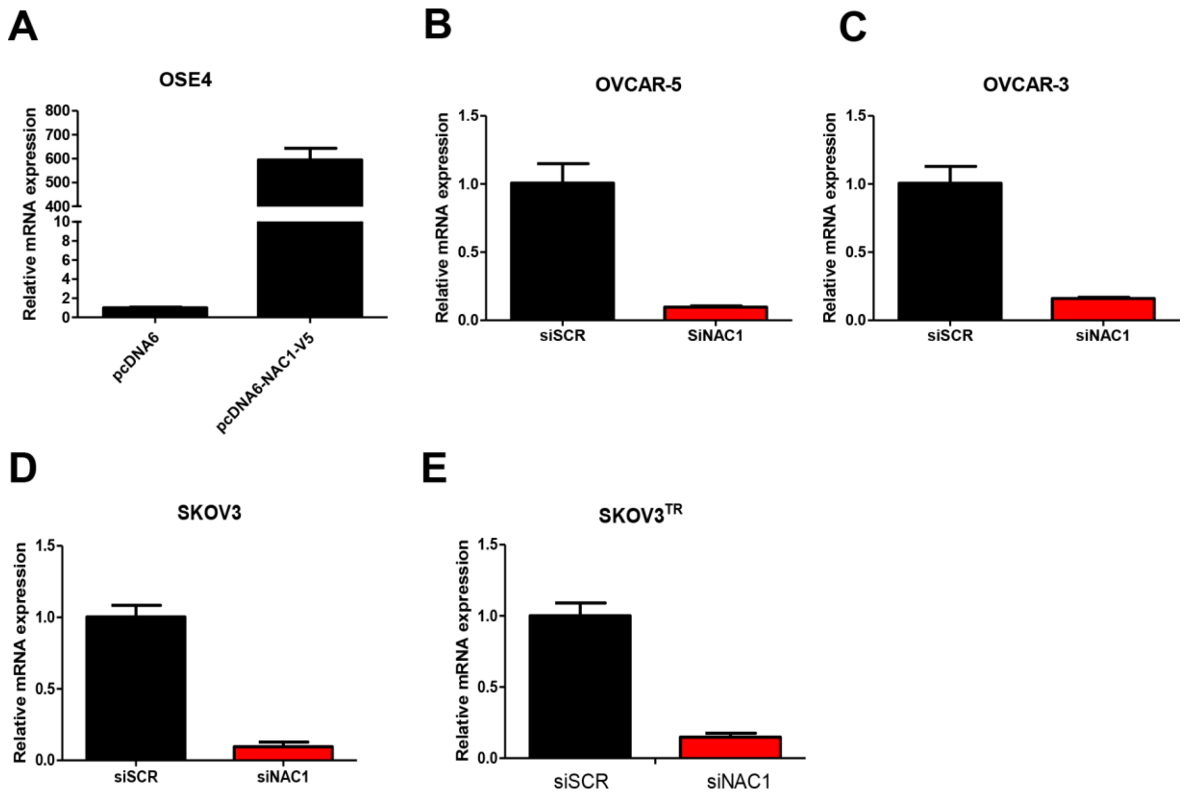

Supplementary Figure 2. NAC1 expression efficiencies determined. NAC1 expression was assessed by qPCR in OSE4 48 h after NAC1-V5 transfection (**A**), and in OVCAR-5 (**B**), OVCAR-3 (**C**), SKOV3 (**D**), and SKOV3TR (**E**) after transfection of NAC1-targeting siRNA.
